# Evaluating Muscular and Cardiovascular Responses to Isometric Handgrip Exercise Among Diabetics and Non-diabetics in a Tertiary Care Center

**DOI:** 10.7759/cureus.84716

**Published:** 2025-05-23

**Authors:** Nishanth Murugan, B Jaya, Janarthanan S, Vasanth Murugan

**Affiliations:** 1 Physiology, Karpaga Vinayaga Institute of Medical Sciences and Research Centre, Chengalpattu, IND; 2 Physiology, Government Tiruvannamalai Medical College, Tiruvannamalai, IND; 3 Physiology, Madras Medical College, Chennai, IND

**Keywords:** blood pressure recovery, cardiovascular response, heart rate recovery, isometric handgrip strength, type 2 diabetics

## Abstract

Introduction: Diabetes mellitus is a chronic metabolic disorder that impairs glucose metabolism, associated with cardiovascular and muscular complications. Research indicates that diabetes may alter heart rate and blood pressure responses, affecting recovery dynamics after physical exertion. Isometric handgrip exercises are a practical method for evaluating muscular strength and cardiovascular function. It provides valuable insights into muscle function and cardiovascular health, particularly in a controlled setting, thereby facilitating the execution of this study.

Aim: To evaluate isometric handgrip strength among diabetic and non-diabetics and to assess the cardiovascular response after isometric handgrip exercise between diabetic and non-diabetic individuals.

Materials and methodology: It is a cross-sectional study involving two groups of male participants aged between 30 to 50 years; a control group of 50 healthy volunteers, and a study group of 50 individuals with type 2 diabetes of less than five years duration under oral hypoglycemic drugs. Isometric strength and endurance were assessed using a handgrip dynamometer. Heart rate and blood pressure were recorded at rest, immediately after the exercise, and at two, and three minutes post-exercise. Data on isometric strength and cardiovascular recovery parameters were compared between the study and control group and analyzed using IBM SPSS Statistics for Windows, Version 28 (IBM Corp., Armonk, NY, USA).

Results: In diabetic individuals baseline isometric handgrip strength, Tmax (kg) (Mean ± SD (30.94 ± 7.7)) was significantly decreased compared to non-diabetics (Mean ± SD (38.62 ± 4.39)) with p<0.001. Both groups showed similar immediate cardiovascular responses to the exercise. However, diabetic individuals demonstrated slower heart rate and blood pressure recovery after exercise compared to non-diabetics with p<0.001.

Conclusion: A decrease in baseline isometric handgrip strength among diabetics might reflect potential metabolic and structural changes in muscle tissue. Following exercise, the slower recovery of cardiovascular responses that occurred in diabetic individuals may be due to disturbances in the autonomic system. These findings suggest that adjusting exercise prescriptions for diabetics can improve cardiovascular health management and hence the quality of life.

## Introduction

Diabetes mellitus is a chronic metabolic disorder characterized by impaired glucose metabolism, which leads to numerous systemic complications, including cardiovascular and muscular dysfunction [1,2]. The chronic nature of diabetes often results in autonomic nervous system disturbances, significantly affecting the body’s responses to physical exertion [3]. One of the most important physiological responses that may be altered in diabetic individuals is heart rate recovery (HRR) and blood pressure recovery after exercise.

HRR refers to the rate at which the heart returns to its baseline rate following physical activity, while blood pressure recovery denotes how quickly blood pressure normalizes post-exercise [4]. Both recovery measures are crucial indicators of autonomic function and cardiovascular health, with slower recovery times being associated with higher risks of cardiovascular events. Impaired recovery dynamics, particularly in diabetic patients, may be indicative of autonomic dysfunction, contributing to the increased cardiovascular morbidity and mortality observed in this population. Furthermore, diabetes contributes to muscular complications, which can further compromise exercise tolerance and recovery.

Isometric handgrip exercises are a practical and effective method for evaluating muscular strength and cardiovascular function [5,6]. This simple yet valuable test allows for the assessment of muscle endurance, strength, and cardiovascular health in a controlled and reproducible manner. Handgrip strength has been widely recognized as a reliable indicator of overall muscular health, and recent studies suggest that it may also reflect cardiovascular health, particularly in terms of autonomic function and the efficiency of the cardiovascular system during exertion [7-9].

The benefits of isometric handgrip exercises lie in their ability to provide insights into muscle function and cardiovascular responses without the need for complex equipment, making it an accessible tool for both clinical and research settings [6,7]. Moreover, it has been shown that grip strength correlates with various health outcomes, including morbidity and mortality, highlighting its significance in evaluating both muscular and cardiovascular health. Given its practical nature and relevance, isometric handgrip exercises are increasingly employed in studies investigating the interplay between physical strength, cardiovascular function, and chronic conditions such as diabetes, hypertension, and heart disease [9,10].

The primary objective of this study is to evaluate isometric handgrip strength among diabetics and non-diabetics and to assess and compare the cardiovascular responses following isometric handgrip exercise between diabetic and non-diabetic individuals.

## Materials and methods

This study was conducted with the approval (Approval No: ECR/774/INST/TN/2015) of the Institutional Ethical Committee at the Research Laboratory of the Physiology Department in Chengalpattu Medical College, a tertiary care center located in Chengalpattu. Ethical clearance was obtained on September 13, 2019, and the study was carried out over a six-month period from July 2020 to December 2020. It was an analytical cross-sectional study involving 100 male participants aged between 30 to 50 years. The study group consisted of 50 individuals who were diagnosed with type 2 diabetes mellitus based on World Health Organization (WHO) criteria for less than five years and without active complications and who were on oral hypoglycemic drugs [11]. The control group comprised 50 age-matched non-diabetic individuals. Sample size estimation was done based on the formula for comparing two independent means with equal variances. Individuals with cardiorespiratory disorders, musculoskeletal or neurological disorders, upper limb injuries or deformities, smokers, alcoholics, and trained athletes were excluded from our study.

History-taking and clinical examination procedures

Subjects were selected based on inclusion and exclusion criteria after getting written informed consent. The procedure and nature of the study were explained to all the subjects in their native language. Personal information, sociodemographic details, and a brief history of present and past illness were taken. Personal history regarding smoking, alcohol, dietary habits, and physical activity were obtained.

After the history-taking general examination, a clinical examination of the cardiovascular system, respiratory system, and central nervous system was done. Vitals such as blood pressure and pulse rate were recorded using the Omron HEM 7124 fully automatic digital blood pressure monitor (Omron Healthcare Co. Ltd., Kyoto, Japan) in the dominant arm in a sitting posture. The pulse rate was also determined by palpating the radial artery to assess the quality of the pulse, temperature, and respiratory rate.

Anthropometric measurements and blood glucose estimation

Anthropometric measurements such as height and weight were measured to calculate body mass index. Under aseptic precautions, blood was drawn from the median cubital vein by venepuncture, and the blood was transferred to the corresponding vacutainer for estimating the fasting and post-prandial blood glucose. Fasting blood glucose was estimated by the hexokinase method.

Grip strength measurement and cardiovascular response

Isometric strength was assessed using the handgrip Baseline Smedley Spring Dynamometer (Fabrication Enterprises, White Plains, NY, USA). After giving a proper demonstration, the subjects were seated in a straight-back chair with their feet flat on the floor. The shoulder was adducted and neutrally rotated, the elbow flexed at 90 degrees, and the forearm in a neutral position with the wrist between 0-30 degrees extension and between 0-15 degrees ulnar deviation. The subjects were instructed to hold the dynamometer with its base resting on the first metacarpal (heel of the palm) and the handle positioned across the middle of the four fingers. They then squeezed the dynamometer with maximum effort for three trials, with a one-minute rest between each trial. The maximum effort did not exceed five seconds for each trial. The maximum voluntary contraction was taken among three trials and it is considered as muscle strength (Tmax in kg).

After five minutes of rest, the subjects were asked to squeeze and hold the dynamometer at 30% maximum voluntary contraction for three minutes. Heart rate and blood pressure were recorded immediately after exercise, and at two minutes and three minutes post-exercise to assess the cardiovascular response [12,13].

Statistical analysis

Data were collected and compared between the groups and analyzed using IBM SPSS Statistics for Windows, Version 28 (IBM Corp., Armonk, NY, USA). The unpaired t-test was used for mean comparisons among study and control groups and repeated measures ANOVA test was used to assess change in blood pressure and heart rate immediately after exercise and at two minutes and three minutes post-exercise within groups. A p-value of <0.05 was considered statistically significant.

## Results

Baseline parameters such as fasting blood sugar (FBS) and post-prandial blood sugar (PPBS) are tabulated in Table 1. The mean and standard deviation of FBS and PPBS were significantly increased (p<0.001) among diabetics when compared to non-diabetics. Mean and standard deviation of age and body mass index (BMI) were found to be not statistically significant and comparable.

**Table 1:** Baseline parameters among study and control group Unpaired t test, p-value of <0.05 was considered statistically significant FBS: fasting blood sugar, PPBS: post-prandial blood sugar

Parameters	Study group (N=50) Mean ± SD	Control group (N=50) Mean ± SD	p-value
Age in years	44.86 ± 4.61	44.13 ± 4.6	0.43
Duration of diabetes	3.5 ± 1.04	N/A	N/A
BMI (Kg/m2)	25.22 ± 3.19	25.26 ± 3.5	0.95
FBS mg/dl	125.84 ± 32.5	75.28 ± 9.66	<0.001
PPBS mg/dl	196.96 ± 57	100.32 ± 7.14	<0.001

Cardiovascular parameters such as heart rate (HR), systolic blood pressure (SBP), and diastolic blood pressure (DBP) are tabulated in Table 2. The mean and standard deviation of HR, SBP, and DBP at rest were not statistically significant and these parameters were comparable among the study and control groups. 

**Table 2:** Cardiovascular parameters at rest among study and control group Unpaired t-test, p-value of <0.05 was considered statistically significant HR: heart rate, SBP: systolic blood pressure, DBP: diastolic blood pressure

Parameters	Study group (N=50) Mean ± SD	Control group (N=50) Mean ± SD	t-value	p-value
HR (beats/min)	85.96 ± 8.04	85.12 ± 4.92	0.63	0.508
SBP (mmHg)	118.52 ± 7.8	118.16 ± 3.42	0.29	0.77
DBP (mmHg)	77.68 ± 8.11	77.04 ± 6.01	0.45	0.65

**Figure 1:**
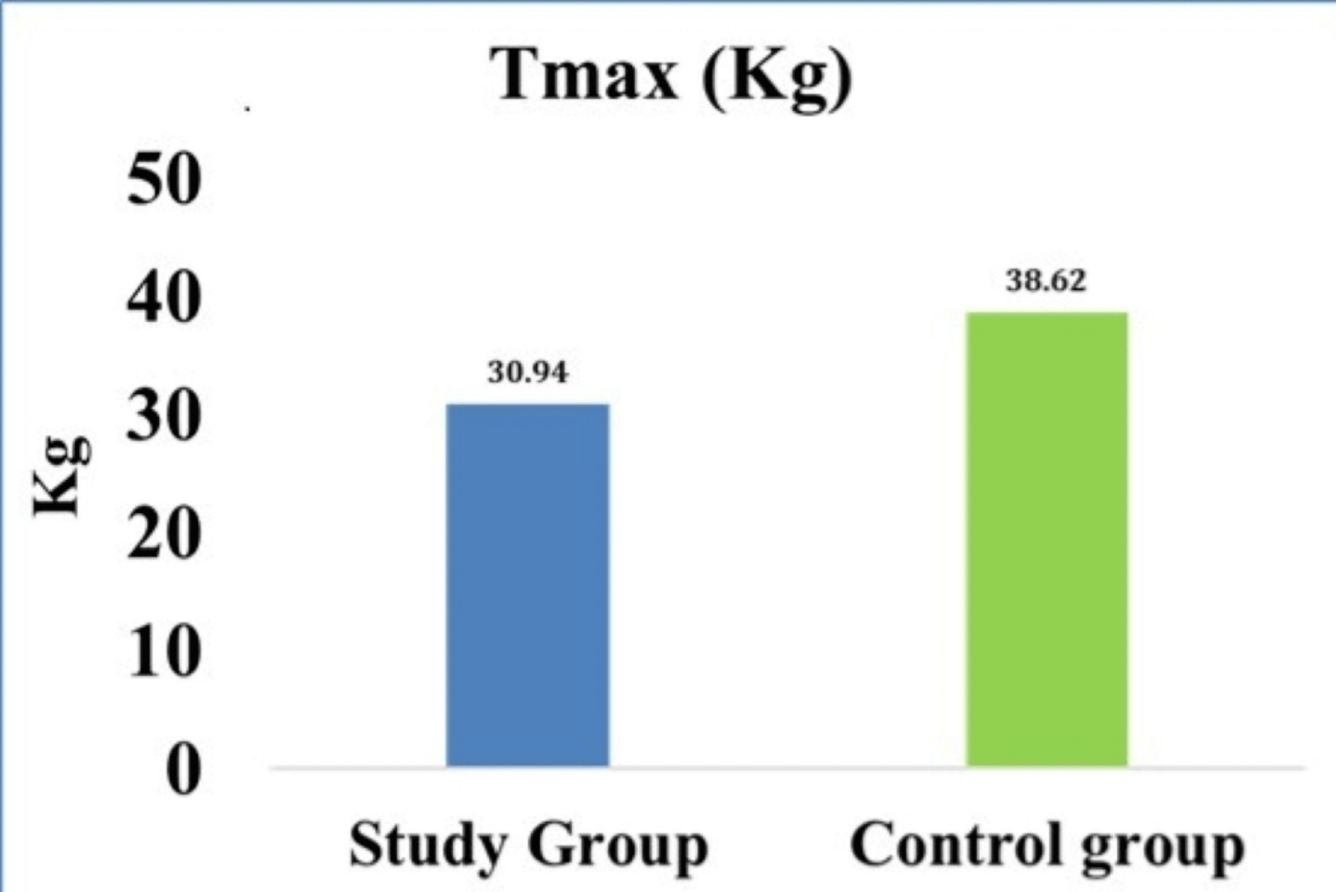
Isometric handgrip strength (Tmax) among study and control group

Isometric handgrip strength (Tmax) among the study and control groups are tabulated in Table 3 and illustrated in Figure 1. The mean and standard deviation of Tmax (kg) is significantly decreased among diabetics compared to non-diabetics with a p-value of <0.001. 

**Table 3:** Isometric handgrip strength (Tmax) among study and control group Unpaired t-test, p-value of <0.05 was considered statistically significant

Parameters	Study group (N=50) Mean ± SD	Control group (N=50) Mean ± SD	t-value	p-value
Tmax (kg)	30.94 ± 7.7	38.62 ± 4.39	6.13	<0.001

**Figure 2:**
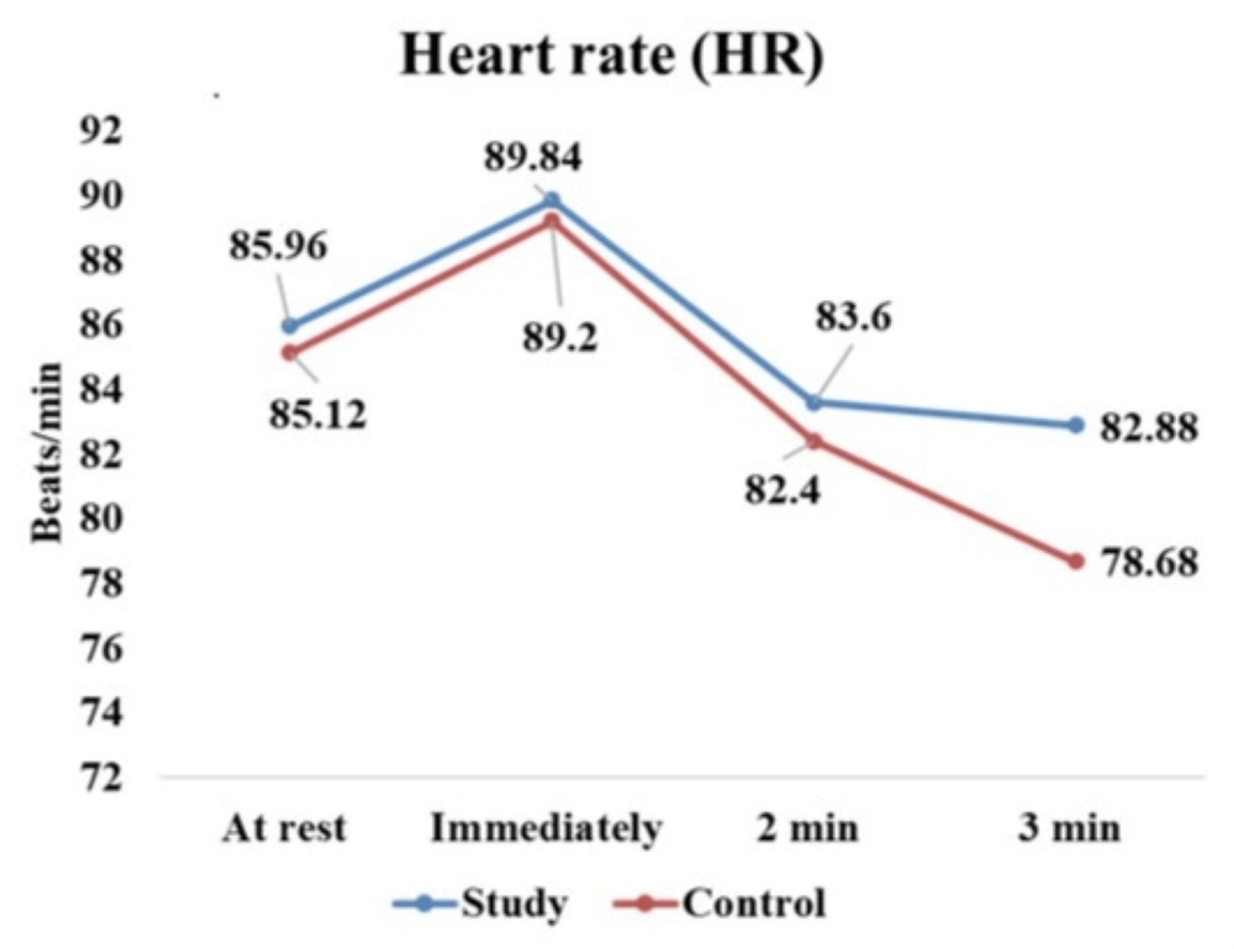
Heart rate (HR) recovery among study and control group

HR recovery among the study and control groups is tabulated in Table 4 and illustrated in Figure 2. The mean and standard deviation of both groups showed similar immediate increases in HR responses to the exercise. However, diabetic individuals demonstrated a significantly lower heart rate recovery after exercise compared to non-diabetics with a p-value <0.001.

**Table 4:** Heart rate (HR) recovery among study and control group Repeated measures ANOVA, p-value of <0.05 was considered statistically significant

Group	Resting HR (beats/min)	HR immediately after exercise (beats/min)	HR 2 min after exercise (beats/min)	HR 3 min after exercise (beats/min)	p-value	F-value
Study (N=50)	85.96 ± 8.04	89.84 ± 12.23	83.6 ± 8.3	82.88 ± 8.46	<0.001	12.212
Control (N=50)	85.12 ± 4.92	89.2 ± 10.50	82.4 ± 10.4	78.68 ±8.90	<0.001	

**Figure 3:**
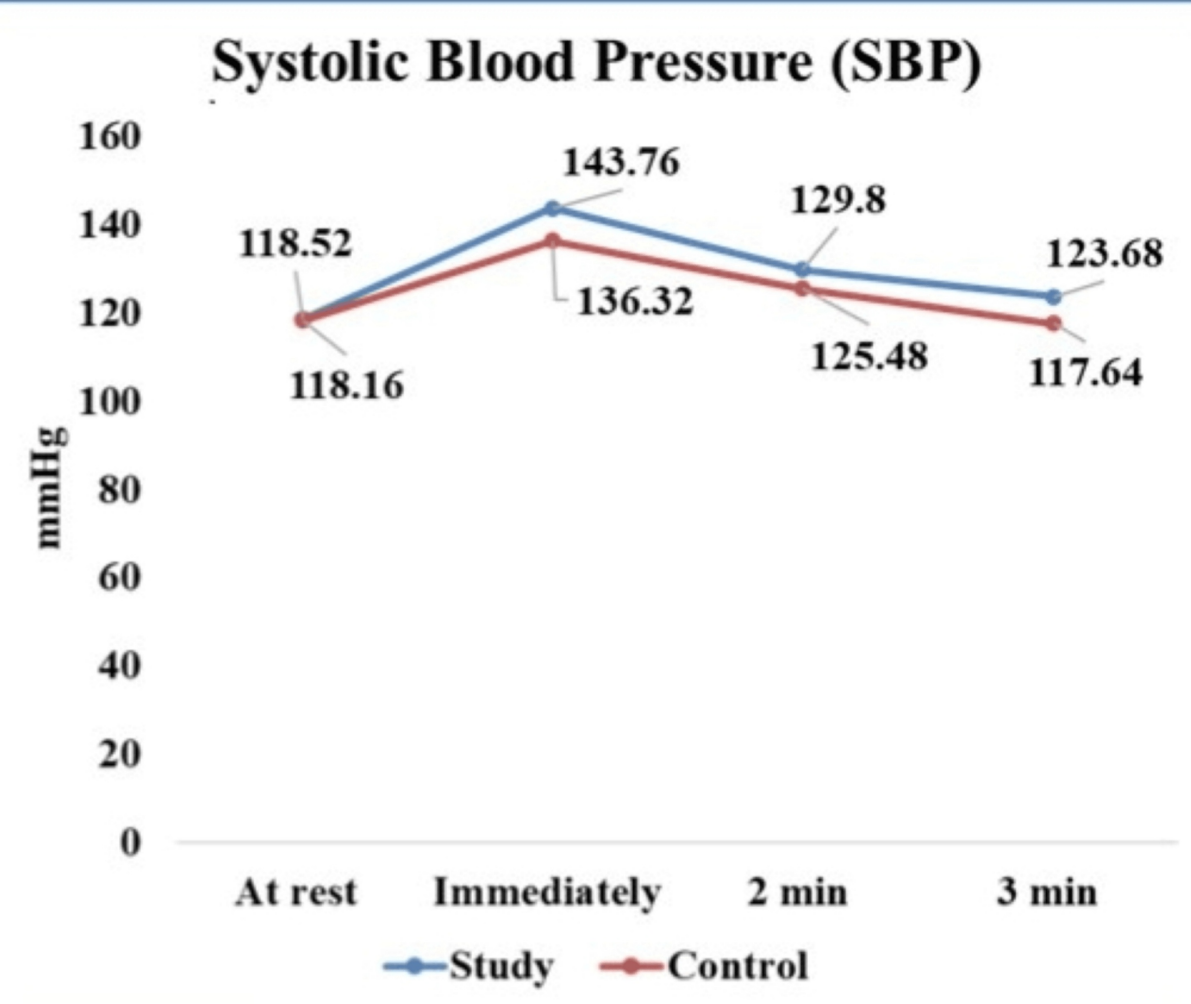
Systolic blood pressure (SBP) recovery among study and control group

SBP recovery among the study and control groups is tabulated in Table 5 and illustrated in Figure 3. The mean and standard deviation of SBP recovery of both groups showed similar immediate increases in SBP responses to the exercise. The study group demonstrated significantly slower recovery of SBP after exercise compared to the control group with a p-value of <0.001. 

**Table 5:** Systolic blood pressure (SBP) recovery among study and control group Repeated measures ANOVA, p-value of <0.05 was considered statistically significant

Group	Resting SBP (mmHg)	SBP immediately after exercise (mmHg)	SBP 2 min after exercise (mmHg)	SBP 3 min after exercise (mmHg)	p-value	F-value
Study (N=50)	118.52 ± 7.8	143.76 ± 17.81	129.8 ± 16.4	123.68 ± 13.8	<0.001	32.80949
Control (N=50)	118.16 ± 3.42	136.32 ± 13.65	125.48 ± 11.71	117.64 ± 11.31	<0.001	

**Figure 4:**
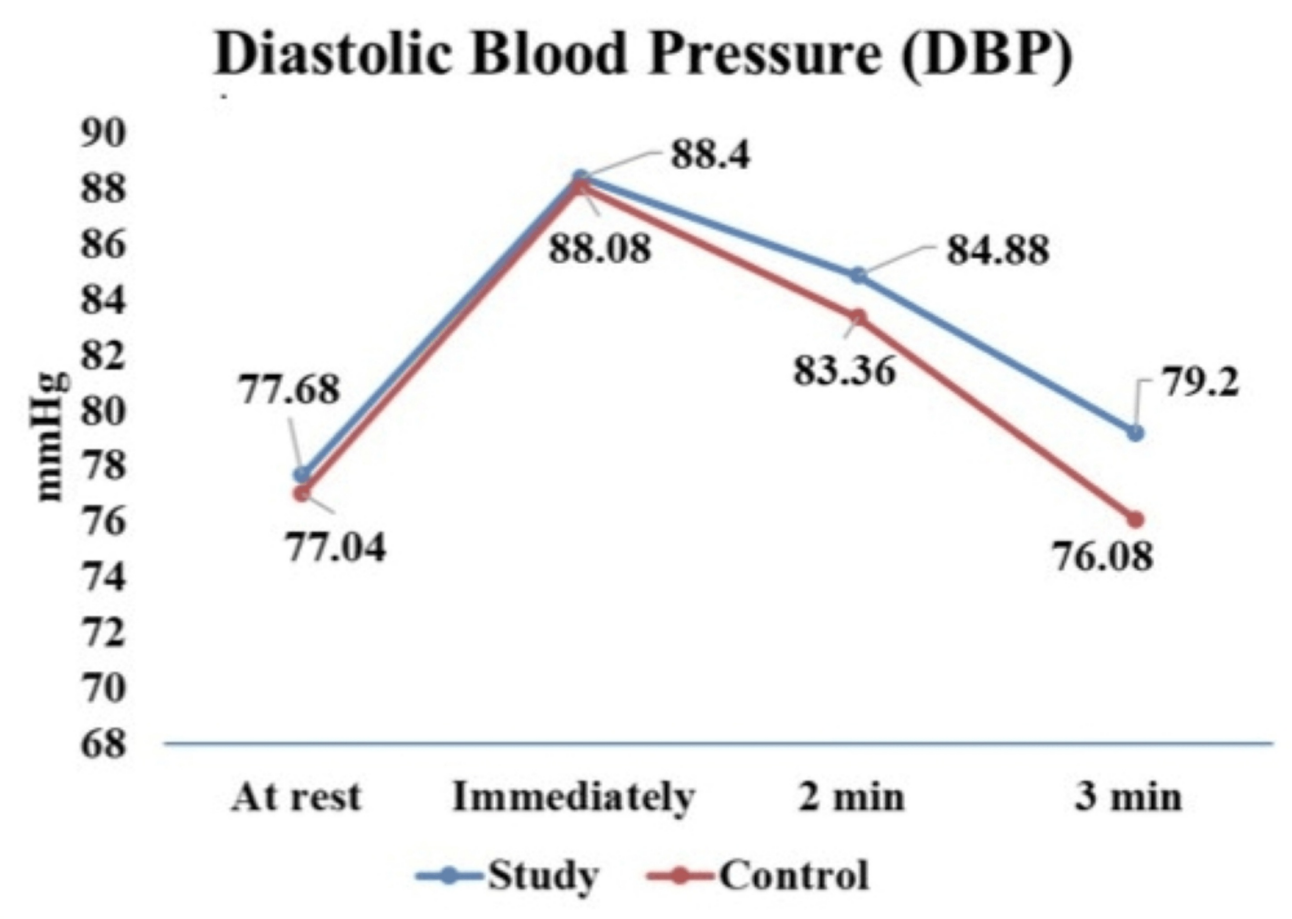
Diastolic blood pressure (DBP) recovery among study and control group

DBP recovery among the study and control groups are tabulated in Table 6 and illustrated in Figure 4. The mean and standard deviation of DBP recovery of both groups showed similar immediate increases in DBP responses to the exercise. The study group demonstrated significantly slower recovery of DBP after exercise compared to the control group with a p-value of <0.001. 

**Table 6:** Diastolic blood pressure (DBP) recovery among study and control group Repeated measures ANOVA, p-value of <0.05 was considered statistically significant

Group	Resting DBP (mmHg)	DBP Immediately after exercise (mmHg)	DBP 2 min after exercise (mmHg)	DBP 3 min after exercise (mmHg)	p-value	F-value
Study (N=50)	77.68 ± 8.11	88.4 ± 11.54	84.88 ± 11.13	79.2 ± 8.04	<0.001	23.45611
Control (N=50)	77.04 ± 6.01	88.08 ± 8.01	83.36 ± 8.12	76.08 ± 10.26	<0.001	

## Discussion

In our study, the age of both the study group (44.86 ± 4.61 years) and the control group (44.13 ± 4.6 years) was found to be comparable. The duration of diabetes in the study group was less than five years. Age is a well-established risk factor in the development, progression, and complications of type 2 diabetes mellitus. As individuals age, there is a decline in insulin sensitivity, pancreatic β-cell function, and lean muscle mass, along with an increase in visceral adiposity and sedentary behavior, all of which contribute to heightened metabolic risk. In our study, participants in the diabetic group were diagnosed between their late 30s and early 40s, indicating that risk factors and early onset of diabetes may begin manifesting even before midlife, highlighting the need for earlier screening and intervention [1].

In our study, the BMI of the study (25.22 ± 3.19) and control groups (25.26 ± 3.5) were found to be comparable, coming under overweight according to the WHO classification of BMI [14]. Overweight individuals may have a high risk of developing insulin resistance, hypertension, and cardiovascular disease.

In our study, a significant decrease in isometric handgrip strength (Tmax) at rest was observed in individuals with diabetes when compared to non-diabetic counterparts. Our results were consistent with Ansari and Ruprai, Uman and Setaiti, Abdul-Ghani and DeFronzo, and Sandhu et al. [15-18]. In their studies, handgrip muscle strength and endurance were decreased among diabetics when compared to non-diabetics [17,18].

The study by Sandhu et al. suggests that a decrease in muscle strength in diabetics is probably due to increased insulin tissue resistance and hyperglycemia, which reduces the number of mitochondria in the muscle cells leading to a decrease in glycogen synthesis [18].

Increased insulin resistance reduces glucose uptake by skeletal muscle due to impaired translocation of GLUT-4 transporters, ultimately leading to decreased adenosine triphosphate (ATP) synthesis. The resulting low ATP availability negatively affects skeletal muscle function and performance. Additionally, insulin resistance and hyperglycemia contribute to elevated levels of systemic inflammatory markers in the circulation, which further impair muscle integrity and function. Moreover, diabetic neuropathy (a common complication of type 2 diabetes mellitus) can disrupt the neural control of muscles, leading to poor coordination and a further decline in muscle strength, particularly grip strength [19,20].

In our study, both groups showed similar immediate increases in heart rate and blood pressure responses to the exercise. Friedman et al. and António et al. state that during isometric exercise, the blood supply to the active muscle is impaired by mechanical compression with static contraction which increases anaerobic metabolism and accumulation of metabolites in active muscle [21,22]. This could increase muscle sympathetic nerve activity which increases cardiovascular parameters in proportion to exercise intensity [21,22].

Moreover, insulin resistance is associated with heightened sympathetic nervous system activity, leading to increased heart rate and blood pressure during and after exercise. Changes in muscle sympathetic nerve activity may be contributing to both decreasing muscle strength and changes in cardiovascular parameters observed in the study [22,23].

In our study, we also observed slower recovery of HR and blood pressure in the study group compared to the control group after the isometric handgrip exercise, with the p-values indicating significant differences between the two groups. Our findings were consistent with other studies that have highlighted similar delays in cardiovascular recovery in individuals with diabetes [24,25].

Slower heart rate recovery is likely due to autonomic neuropathy, a condition that damages the autonomic nervous system and impairs its ability to regulate heart rate during and after exercise. As a result, the heart is less responsive to the stress induced by physical exertion [26].

Moreover, impaired vagal reactivation is one of the key components of autonomic function where a delayed parasympathetic system contributes to the reduced efficiency of post-exercise recovery, making it more difficult for the heart rate to return to baseline levels. In addition, the elevated sympathetic tone can persist after exercise, hindering the normalization of cardiovascular parameters [27].

Similarly, the slower recovery of SBP and DBP observed in our diabetic participants may be attributed to insulin resistance, which leads to endothelial dysfunction and increased arterial stiffness. Reduced nitric oxide availability impairs vasodilation, leading to increased vascular resistance and elevated blood pressure which leads to endothelial dysfunction [28-30].

In addition to that, elevated glucose and insulin levels contribute to structural changes in the arterial wall, increasing stiffness and reducing the ability of blood vessels to expand and contract appropriately, which further hinders the cardiovascular system’s ability to recover swiftly following physical activity [31,32].

Limitations

This study included only male participants, limiting the generalizability to females due to gender-based physiological differences. Being cross-sectional, it captures data at a single time point and cannot assess long-term effects or causality. The sample size, though adequate, could be expanded for stronger statistical power. Participants had type 2 diabetes for less than five years and no complications, so findings may not apply to those with longer disease duration or complications. Additionally, variations in medication, lifestyle, and dietary habits were not controlled, which may have influenced the outcomes.

## Conclusions

The decrease in baseline isometric handgrip strength among diabetics could likely be attributed to metabolic and structural changes in muscle tissue. Following exercise, the slower recovery of cardiovascular responses observed in diabetic individuals may be due to disturbances in the autonomic nervous system. These findings provide critical insights into cardiovascular health disparities in the early stages of diabetes. It highlights the need for targeted interventions to improve autonomic function and vascular responsiveness. Regular exercise, along with lifestyle modifications and consistent medication, can significantly enhance cardiovascular health management, ultimately improving the quality of life for individuals with diabetes.
